# Open Right Hemicolectomy:Lateral to Medial or Medial to Lateral Approach?

**DOI:** 10.1371/journal.pone.0145175

**Published:** 2015-12-31

**Authors:** Pingping Xu, Li Ren, Dexiang Zhu, Qi Lin, Yunshi Zhong, Wentao Tang, Qingyang Feng, Peng Zheng, Meiling Ji, Ye Wei, Jianmin Xu

**Affiliations:** 1 Department of General Surgery, Zhongshan Hospital, Fudan University, Shanghai, China; 2 Department of Endoscopy Center, Zhongshan Hospital, Fudan University, Shanghai, China; Osaka Medical Center for Cancer and Cardiovascular Diseases, JAPAN

## Abstract

**Objective:**

Currently, no published studies have compared the clinical outcomes of the medial-to-lateral approach (MA) and lateral-to-medial approach (LA) for open right hemicolectomy. Thus, the present study aimed to assess whether one of these approaches has any potential benefits over the other.

**Methods:**

A retrospective study was performed of all patients who underwent open right hemicolectomy with pathologically confirmed disease who met the eligibility criteria between June 2008 and June 2012. The population was divided into an MA group and an LA group by propensity scoring. We compared patient demographic and clinical characteristic variables between the two groups and assessed short-term and long-term outcomes.

**Results:**

A total of 450 patients (MA, n = 150; LA, n = 300) were evaluated. The operation time (MA,138.4 minutesvs.LA,166.2 minutes; *P* < .05) and blood loss (MA,52.0mL vs. LA,62.6mL; *P* < .05)were significantly lower in the MA group. No differences in the number of harvested lymph nodes and oncologic outcomes were observed between the two groups. Further subgroup analysis for stage III colon cancer revealed that the MA group had significantly more retrieved lymph nodes (MA,18.8vs. LA,16.0; *P =* .028). There were no differences in other variables between the two groups.

**Conclusions:**

The MA reduced operative time and blood loss compared with the LA. We thus concluded that the MA provided short-term benefits compared with the LA in open right hemicolectomy for right-sided colon cancer.

## Introduction

Colorectal cancer (CRC) is the third most commonly diagnosed cancer in males and the second in females, with over 1.4million new cancer cases and 693,900 deaths estimated to have occurred worldwide [1]. The incidence of colorectal cancer in China is rapidly increasing, and colorectal cancer is the most frequently occurring gastrointestinal cancer in large cities, such as Beijing and Shanghai [2,3]. Among them, right-sided colon cancer represents nearly one-third of all cases, and surgery is the main treatment for this disease [4,5].

The traditional sequence used in open right hemicolectomy begins with lateral-to-medial approach (LA)[6]. With the uptake of minimal access techniques, many laparoscopic surgeons tried to use the medial-to-lateral approach (MA)[7,8,9]. Due to the rigorous indication recommended by the National Comprehensive Cancer Network (Version 2.2015) for colon cancer, many established specialist surgeons continue to perform open surgery with excellent outcomes [10].

Recently, some studies have compared the safety and efficacy of the MA and LA in laparoscopic colorectal surgery, revealing that the MA provides some potential short-term advantages [6,11,12,13,14,15].To our knowledge, no study has compared the MA and LA in open right hemicolectomy. Therefore, the objective of the present study was to evaluate the potential benefits of one of the approaches in a consecutive series of patients who underwent right open hemicolectomy.

## Patients and Methods

### Study population and patient selection

A total of 450consecutive patients (MA,150 patients; LA,300 patients) who underwent open right hemicolectomy for right-sided colon cancer at Zhongshan Hospital affiliated with Fudan University, Shanghai, between June 2008 and June 2012 were included in our prospectively constructed colorectal cancer (CRC)database. By propensity score analysis,300 patients who underwent right open hemicolectomy were matched with150 patients with the MA. All of the surgeries were performed by one surgical team from the General Surgery department of Zhongshan Hospital affiliated with Fudan University. The approach selected for each patient depended on the choice of chief surgeon. During the learning curve period the surgical team visited Massachusetts general hospital at Harvard university in USA and AmbroiseParé Hospital at Paris V University to learn the MA and LA respectively. Therefore both of the approaches performed in our study were considered to be standardized and practiced. Institutional review board approval was obtained from Zhongshan Hospital, which is affiliated with Fudan University for this retrospective analysis. All patients provided written informed consent. All patients with stage II colon cancer with poor prognostic factors and stage III colon cancer received chemotherapy (5-fluorouracil based or capecitabine). The selection criteria for open right hemicolectomy include the following: obstructive colorectal cancer, cancer perforation, stage IV colon cancer, and asynchronous or previous malignancies. Follow-up information was obtained through outpatient visits or telephone inquiries at Zhongshan Hospital, Fudan University. Perioperative clinicopathologic data, morbidity, mortality, and short-term and long-term oncologic outcomes were compared between the MA and LA groups. All patients were assessed preoperatively with fiberoptic colonoscopy; contrast-enhanced chest, abdominal, and pelvic computed tomography; and rectum magnetic resonance imaging. In our study, the final diagnosis was based on pathological morphology and immunohistochemical assessment through surgical specimen and intraoperative biopsy by two experienced pathologists.

### Procedures

For all patients, mechanical preparation of the colon was performed the day before surgery.

The following procedure was used for the medial-to-lateral approach (MA) for open right hemicolectomy: The ileocolic pedicle was identified, exposing the ileocolic vessels. The mesentery surrounding the vessels was transected, exposing a gap between the mesocolon and retroperitoneal fat. This was followed by blunt dissection of the avascular space up to the hepatic flexure with identification of the duodenum and ligation and dissection of the ileocolic vessels and the right branch of the middle colic vessels at their root. After transection of the gastrocolic ligament, the hepatic flexure and lateral attachments of ascending colon were finally mobilized.

The following procedure was used for the lateral-to-medial approach (LA) for open right hemicolectomy: The cecum and terminal ileum were mobilized cephalad. Dissection of the Toldt fascia from the ileocecal junction to the hepatic flexure was followed by blunt dissection to separate the mesocolon from the retroperitoneal fat. After identification of the duodenum, the hepatic flexure and transverse colon were released, thus completing right colon mobilization. Finally, the colon was ligated and anastomosis was performed.

### Propensity score matching

The PSM approach for the present study proceeded in two steps. First, the propensity score of each patient who underwent open right hemicolectomy was calculated based on a logistic regression model, including age, gender, body mass index (BMI), American Society of Anesthesiologists(ASA) score, primary tumor location, largest size of primary tumor, histological type, differentiation, tumor site, preoperative chemotherapy regimen, preoperative CEA, previous abdominal surgery,retrieved lymph nodes. These variables were chosen empirically based on factors we believed to be important contributors to the operative difficulty risk of complications/mortalities and differences in oncologic outcomes. In the second step, the MA group patients were matched 1:2 based on the closest propensity score to LA group patients. The process of matching based on the propensity score yields a matched sample (1:2) that is better balanced in the covariates included in the selection model.

### Statistical Methods

Statistical analyses were performed using the SPSS statistical package (version 16.0;SPSS; Chicago, IL). All P values were two-sided, and the significant level was specified as P<0.05 in all analyses. Summary statistics were obtained using established methods and were represented as percentages or mean values with standard deviation. The baseline characteristics and perioperative and long-term oncologic outcomes of the matched data were compared using an independent-sample t test (or Mann-Whitney U test) for continuous variables and chi-square analysis or Fisher’s exact test for categorical variables. Survival rates were estimated using the Kaplan-Meier method, and differences in survival curves were compared using the log-rank test.

## Results

### Clinicopathologic Characteristics

From June 2008 to June 2012, a total of 450 patients were studied, with the following distribution:150 patients in the MA group (33.3%) and 300 patients in the LA group (66.7%). There were no significant differences in age(*P* = .56), sex(*P* = .51), BMI(*P* = .84), American Society of Anesthesiologists grade(*P* = .78), tumor location(*P* = .48), previous abdominal surgery(*P* = .89), preoperative chemotherapy(*P* = .69) or preoperative carcinoembryonic antigen (*P* = .67) between the two groups, as shown in Table 1.

**Table 1:** Baseline Patient Demographics and Clinical Characteristics.

Characteristics	LA Group (n = 300)		MA Group (n = 150)		p-Value
	No.	%	No.	%	
**Age(years)**					.56
Median	62.5		64.0		
Range	24–84		26–86		
**Sex**					.51
Male	160	53.3	75	50.0	
Female	140	46.7	75	50.0	
**BMI(weigh/height** ^**2**^ **)**					.84
Underweight	31	10.3	12	8.0	
Normal	194	64.7	98	65.3	
Overweight	46	15.3	26	17.3	
Obese	29	9.7	14	9.4	
**ASA grade**					.78
I	61	20.3	31	20.7	
II	238	79.4	119	79.3	
III	1	.3	0	.0	
**Site of cancer**					.48
Ileocecum	70	23.3	28	18.7	
Ascending colon	151	50.3	73	48.7	
hepatic flexure of colon	67	22.3	42	28.0	
Right side of transverse colon	12	4.1	7	4.6	
**Preoperative chemotherapy**					.69
FOLFOX	185	61.7	101	63.1	
XELOX	36	12.0	15	9.4	
Others	79	26.3	44	27.5	
**Previous abdominal surgery**	92	30.7	45	30.0	.89
**Preoperative CEA, ng/ml**					.67
<5	202	67.3	136	65.3	
≥5	98	32.7	68	34.7	

Abbreviations: BMI:body mass index;ASA: American Society of Anesthesiologists, CEA:carcinoembryonic antigen; LA: lateral-to-medial approach; MA: medial-to-lateral approach.

### Perioperative complications and short-term outcomes

After evaluating surgical perioperative outcomes and complications, we found that patients in the MA group had a significantly shorter operative time (MA,138.4 minutes vs. LA,166.2 minutes; *P <* .05) and less blood loss (MA,52.0 mL vs. LA,62.6 mL; *P <* .05) than the LA group. There were no significant differences in first flatus POD (*P* = .46), time to liquid diet (*P* = .07), postoperative hospital stay (*P* = .27), complications during surgery (*P* = .86), minor postoperative complications (*P* = .85), major postoperative complications (*P* = .81), or mortality within 30 days after surgery (*P* = .48), as shown in Table 2.

**Table 2:** Perioperative outcomes and complications.

Characteristics	LA Group (n = 300)	MA Group (n = 150)	p-Value
**Blood loss, mL**			.004
Median	62.6	52.0	
SD	42.7	19.8	
**Duration of surgery, minutes**			.000
Mean	166.2	138.4	
SD	27.9	12.7	
**First flatus POD Days**			.46
Mean	2.6	2.5	
SD	0.6	0.7	
**Time to liquid diet, days**			.07
Mean	3.9	3.6	
SD	2.0	0.7	
**Postoperative hospital stay, days**			.27
Mean	8.1	7.7	
SD	4.2	2.1	
**Complications during surgery**			.86
Intraoperative hemorrhage (>500 mL)	1	0	
Pulmonary insufficiency	1	1	
Cardiac insufficiency	2	1	
Poor visualization	3	2	
**Minor postoperative complications**			.85
Chyle leakage	24	12	
Wound infection	4	2	
Urinary tract infection	1	1	
Chest infection	2	0	
Paralytic ileus (IV fluids>7 days)	3	2	
**Major postoperative complications**			.81
Respiratory failure requiring ventilation	1	0	
Renal failure requiring dialysis	1	0	
Cardiac failure, myocardial infarction	1	0	
Anastomotic leakage	2	2	
Bowel obstruction requiring second surgery	1	1	
Abdominal wall dehiscence requiring surgery	2	1	
**Mortality within 30days after surgery**	1	0	.48

POD postoperative day

### Postoperative Pathologic Results

The histologic differentiation of the tumor (*P* = .98), pT stage (*P* = .48), pN stage (*P* = .84), TNM stage (*P* = .65), morphology (*P* = .37) and maximum tumor diameter (*P* = .23) did not differ significantly between the two groups. In addition, there were no significant differences in the median number of retrieved lymph nodes (MA, 17.0vs. LA, 16.9, *P* = .86) or number of positive lymph nodes (MA, 1.0vs. LA, 0.7, *P* = .12) between the two groups, as shown in Table 3. Further subgroup analysis for stage II and stage III colon cancer revealed that for stage II cancer, there was no difference in number of harvested lymph nodes (MA, 17.0vs. LA, 17.6; *P* = .55) between the two groups. However, for stage III cancer, the MA group had significantly more retrieved lymph nodes (MA, 18.8vs. LA, 16.0; *P* = .028) and positive lymph nodes (MA, 3.4vs. LA, 2.2; *P* = .025), as shown in Table 4.

**Table 3:** Pathology Data.

Characteristics	LA Group(n = 300)		MA Group(n = 150)		p-Value
	No.	%	No.	%	
**pT stage**					.48
1	15	5.0	6	4.0	
2	21	7.0	8	5.3	
3	13	4.3	3	2.0	
4	251	83.7	133	88.7	
**pN stage**					.84
0	205	68.3	106	70.7	
1	65	21.7	29	19.3	
2	30	10.0	15	10.0	
**No. of retrieved lymph nodes**					.86
Mean	16.9		17.0		
SD	8.3		7.6		
**No. of positive lymph nodes**					.12
Mean	0.7		1.0		
SD	1.4		2.9		
**TNM stage**					.65
I	34	11.3	14	9.3	
II	171	57.0	92	61.3	
III	95	31.7	44	29.4	
**Differentiation**					.98
Well or moderate	245	81.7	120	80.0	
Poor	55	18.3	30	20.0	
**Morphology**					.37
Protuberant mass	105	35.0	41	27.3	
Ulcerative mass	176	58.7	100	66.7	
Infiltrating mass	6	2.0	2	1.3	
Fungating mass	13	4.3	7	4.7	
**Maximum tumor diameter, cm**					.23
<5	124	41.3	71	47.3	
≥5	176	58.7	79	52.7	

**Table 4:** Pathology Data for Stage II and Stage III colon caner.

Pathology Data for Stage II colon caner			
Characteristics	LA Group(n = 171)	MA Group(n = 92)	p-Value
**No. of retrieved lymph nodes**			.55
Mean	17.6	17.0	
SD	7.9	8.4	
Pathology Data for Stage III colon caner			
Characteristics	LA Group(n = 95)	MA Group(n = 44)	
**No. of retrieved lymph nodes**			.028
Mean	16.0	18.8	
SD	6.9	7.5	
**No. of positive lymph nodes**			.025
Mean	2.2	3.4	
SD	1.7	4.7	

### Long-term outcomes

**Fig 1:**
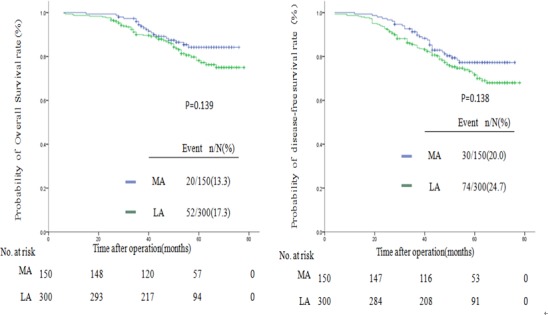
Kaplan-Meier estimates of survival between the MA group and the LA group. Kaplan-Meier analysis of OS(*P* = .139)and DFS(*P* = .138) between the MA group and the LA group.

Follow-up information until October 2014 was obtained for the 450 patients. Using the Kaplan-Meier method, the 5-year overall survival (OS) rates of the MA and LA groups were 84% and 78% (*P* = .139), respectively, and the 5-year disease-free survival (DFS) rates were 76% and 65% (*P* = .138), respectively, as shown in Fig 1.

**Fig 2:**
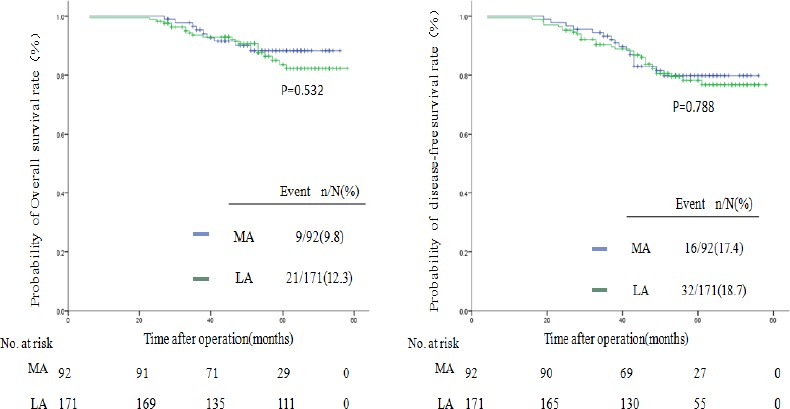
Kaplan-Meier estimates of survival between the MA group and the LA group for stage II colon cancer. Kaplan-Meier analysis of OS(*P* = .532) and DFS(*P* = .788).

**Fig 3:**
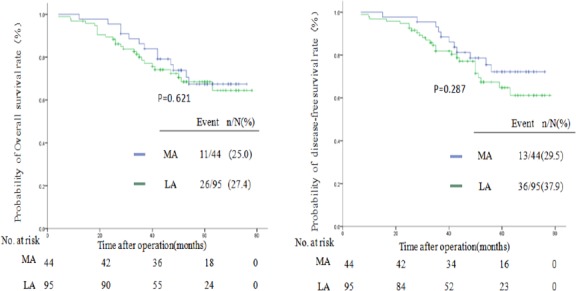
Kaplan-Meier estimates of survival between the MA group and the LA group for stage III colon cancer. Kaplan-Meier analysis of OS(*P* = .621) and DFS(*P* = .287).

In subgroup analysis of stage II and stage III colon cancer, the 5-year OS of both stage II right-sided colon cancer (MA, 88%vs. LA 82%; *P* = .532) and stage III colon cancer (MA,72%vs. LA 62%; *P* = .621) were comparable between the MA and LA groups. Meanwhile, no difference was found in the 5-year DFS rates for stage II right-sided colon cancer (MA, 79%vs. LA 78%; *P* = .788) or stage III colon cancer (MA, 71%vs. LA 60%; *P* = .287), as shown in Figs 2 and 3.

## Discussion

Our retrospective study demonstrated the feasibility and safety of MA for open right hemicolectomy. The MA reduced operative time and blood loss compared with the LA. Although the MA for stage III colon cancer was associated with more retrieved lymph nodes compared with the LA, the long-term oncologic outcomes between the groups were comparable. We thus concluded that the MA has an outcome similar to the LA but has some short-term advantages.

With the development of surgical skills and instrumental technology, some laparoscopic surgeons have performed the right hemicolectomy with good results using the MA but not the LA in conventional open right hemicolectomy[14,16,17,18,19]. Some studies have compared the two approaches in terms of safety, efficacy and oncologic outcomes in laparoscopic surgery, reporting that the MA showed some potential short-term advantages[19]. Moreover, an International Consensus Conference sponsored by the European Association of Endoscopic Surgery (EAES) consensus issued a statement the MA is the preferred method during laparoscopic colectomy[20]. Nonetheless, there has been no literature published that discusses the clinical outcomes of the two approaches in open right hemicolectomy. We believe that our study is significant because it represents the first retrospective study comparing the two approaches in open right hemicolectomy.

There are five published research studies comparing the MA and LA in patients who underwent laparoscopic right hemicolectomy. In a retrospective comparative study lacking an LA group, Pagazzi et al [14] supported the concept that the MA in laparoscopic right hemicolectomy resulted in shorter operative time, lower conversion rates, and overall complication rates. Liang et al [13] published the results of a phase II clinical trial regarding the utilization of the MA for right-sided colon cancer. The authors encouraged the use of MA for patients requiring a laparoscopic right hemicolectomy. Rotholtz et al [6] reported that the MA significantly reduced the operation time and time to intestinal recovery. Yan et al [11] published results showing that the MA resulted in a shorter operative time and less blood loss in laparoscopic right hemicolectomy. That study was the only randomized prospective trial comparing the MA and the LA in patients undergoing laparoscopic right hemicolectomy. However, Ballantyne et al [18]showed that the MA achieved the same outcomes as the LA in laparoscopic right hemicolectomy, but their study was a retrospective comparative study with a limited number of patients. Our study revealed a significantly shorter operative time and less blood loss in the MA group. Possible reasons for this were as follows: a).with the MA, it was easy for the surgeon to keep the dissection at the bloodless plane between the mesocolon and the retroperitoneum and to reduce blood loss accordingly[6];b).less amount of bleeding was expected with MA during mobilization of the colon when ligating the vessels primarily[11]; c).in some rare conditions in which the colonic tumor coexists with a colonic inflammatory process (e.g., diverticulitis),the LA might be difficult and dangerous because the LA is blurred during the divisions[19]. Our results of operative time and blood loss are in agreement with reported operation times in published studies.

Recently, some studies have investigated the effect of the harvested lymph node number on oncologic outcome and have shown that an increased lymph node yield after surgery for stage II and III colon cancer is associated with survival benefits[21,22]. We found no significant differences in terms of the number of retrieved lymph nodes. In the further subgroup analysis for stage II or stage III colon cancer, there was also no difference in median numbers of harvested lymph nodes for stage II colon cancer. In contrast, the number of harvested lymph nodes of stage III colon cancer patients was increased in the MA group compared with the LA group. A few studies, including two prospective randomized controlled trials comparing MA and LA in laparoscopic colorectal surgeries, have also shown no significant difference in number of harvested lymph nodes between the two groups [18,19].We speculated that the differences in number of retrieved lymph nodes for stage III colon cancer might result from the more efficient technique and superior surgical field in the MA group. However, whether MA can achieve more lymph nodes for stage III colon cancer remained to be confirmed.

With respect to oncologic outcomes, the 5-year OS and 5-year DFS did not differ significantly between the two groups either in the overall comparison or in the subgroup analysis for stage II or stage III colon cancer. The 5-year OS in our study is in agreement with that of recent studies reporting that approximately 47.4%-80% of patients with open right hemicolectomy will survive five or more years [9,13,23,24,25]. The 5-year DFS mirrors results from other studies reporting that these patients actually achieved a DFS of nearly 50–84% [13,23,24,25]. The number of retrieved lymph nodes of stage III colon cancer patients was more in the MA group. However, the 5-year OS and 5-year DFS for stage III right-sided colon cancer between the two groups showed no differences.

The appropriate surgical approach is an important part of the standardization of surgery, which can better expose the surgical field [12]. We believe that our study is helpful for the standardization of open right hemicolectomy, as well as in shortening the learning curve and achieving a better therapeutic effect. Although our study revealed that open right hemicolectomy with the MA has many advantages, we believe that the MA is applicable only for patients with resectable cancer, and when it is not clear whether the lesions can be resected, the LA should be used. Otherwise, if the surgeon applies a medial approach and ligates the major vessels in the case of unresectable lesions, causing ischemia and necrosis at the devascularized colonic segment, the surgeon will face an awkward dilemma.

There are some limitations of this study. First, our study was not a well-designed, multicenter, randomized controlled trial, and our follow-up time was short. Second, in general, the surgeons initially performed the open right hemicolectomy using the LA, and the MA was used during the surgeons’ more advanced learning period, and this could have generated a bias as a result of the learning curve.

## Conclusions

In the current limited retrospective study, with shorter operative time and less blood loss, we concluded that the MA provided short-term benefits compared with the LA in open right hemicolectomy for right-sided colon cancer. The MA might be an improvement over the previously used LA approach for surgeons performing open right hemicolectomy. However, it is necessary to conduct well-designed, multicenter, prospective randomized controlled trials to allow for a more convincing evaluation.

## References

[1] Torre LA, Bray F, Siegel RL, Ferlay J, Lortet-Tieulent J, et al (2015) Global cancer statistics, 2012. CA Cancer J Clin 65: 87–108. 10.3322/caac.21262 25651787

[2] Gu J, Chen N (2013) Current status of rectal cancer treatment in China. Colorectal Dis 15: 1345–1350. 10.1111/codi.12269 23651350

[3] Dexiang Z, Li R, Ye W, Haifu W, Yunshi Z, et al (2012) Outcome of patients with colorectal liver metastasis: analysis of 1,613 consecutive cases. Ann Surg Oncol 19: 2860–2868. 10.1245/s10434-012-2356-9 22526903

[4] Siegel R, Desantis C, Jemal A (2014) Colorectal cancer statistics, 2014. CA Cancer J Clin 64: 104–117. 10.3322/caac.21220 24639052

[5] Lee GH, Malietzis G, Askari A, Bernardo D, Al-Hassi HO, et al (2015) Is right-sided colon cancer different to left-sided colorectal cancer?—a systematic review. Eur J Surg Oncol 41: 300–308. 10.1016/j.ejso.2014.11.001 25468456

[6] Rotholtz NA, Bun ME, Tessio M, Lencinas SM, Laporte M, et al (2009) Laparoscopic colectomy: medial versus lateral approach. Surg Laparosc Endosc Percutan Tech 19: 43–47. 10.1097/SLE.0b013e31818e91f3 19238066

[7] Rondelli F, Trastulli S, Avenia N, Schillaci G, Cirocchi R, et al (2012) Is laparoscopic right colectomy more effective than open resection? A meta-analysis of randomized and nonrandomized studies. Colorectal Dis 14: e447–e469. 10.1111/j.1463-1318.2012.03054.x 22540533

[8] Green BL, Marshall HC, Collinson F, Quirke P, Guillou P, et al (2013) Long-term follow-up of the Medical Research Council CLASICC trial of conventional versus laparoscopically assisted resection in colorectal cancer. Br J Surg 100: 75–82. 10.1002/bjs.8945 23132548

[9] Buunen M, Veldkamp R, Hop WC, Kuhry E, Jeekel J, et al (2009) Survival after laparoscopic surgery versus open surgery for colon cancer: long-term outcome of a randomised clinical trial. Lancet Oncol 10: 44–52. 10.1016/S1470-2045(08)70310-3 19071061

[10] Kennedy RH, Francis EA, Wharton R, Blazeby JM, Quirke P, et al (2014) Multicenter randomized controlled trial of conventional versus laparoscopic surgery for colorectal cancer within an enhanced recovery programme: EnROL. J Clin Oncol 32: 1804–1811. 10.1200/JCO.2013.54.3694 24799480

[11] Yan J, Ying MG, Zhou D, Chen X, Chen LC, et al (2010) [A prospective randomized control trial of the approach for laparoscopic right hemi-colectomy:medial-to-lateral versus lateral-to-medial]. Zhonghua Wei Chang Wai Ke Za Zhi 13: 403–405. 20577914

[12] Ding J, Liao GQ, Xia Y, Zhang ZM, Pan Y, et al (2013) Medial versus lateral approach in laparoscopic colorectal resection: a systematic review and meta-analysis. World J Surg 37: 863–872. 10.1007/s00268-012-1888-2 23254947

[13] Liang JT, Lai HS, Lee PH (2007) Laparoscopic medial-to-lateral approach for the curative resection of right-sided colon cancer. Ann Surg Oncol 14: 1878–1879. 17377832 10.1245/s10434-006-9153-2

[14] Pigazzi A, Hellan M, Ewing DR, Paz BI, Ballantyne GH (2007) Laparoscopic medial-to-lateral colon dissection: how and why. J Gastrointest Surg 11: 778–782. 17562120 10.1007/s11605-007-0120-4

[15] Poon JT, Law WL, Fan JK, Lo OS (2009) Impact of the standardized medial-to-lateral approach on outcome of laparoscopic colorectal resection. World J Surg 33: 2177–2182. 10.1007/s00268-009-0173-5 19669230

[16] Milsom JW, Bohm B, Hammerhofer KA, Fazio V, Steiger E, et al (1998) A prospective, randomized trial comparing laparoscopic versus conventional techniques in colorectal cancer surgery: a preliminary report. J Am Coll Surg 187: 46–54, 54–55. 9660024 10.1016/s1072-7515(98)00132-x

[17] Senagore AJ, Duepree HJ, Delaney CP, Brady KM, Fazio VW (2003) Results of a standardized technique and postoperative care plan for laparoscopic sigmoid colectomy: a 30-month experience. Dis Colon Rectum 46: 503–509. 12682545 10.1007/s10350-004-6590-5

[18] Ballantyne GH, Ewing D, Pigazzi A, Wasielewski A (2006) Telerobotic-assisted laparoscopic right hemicolectomy: lateral to medial or medial to lateral dissection? Surg Laparosc Endosc Percutan Tech 16: 406–410. 17277657 10.1097/01.sle.0000213732.03204.50

[19] Liang JT, Lai HS, Huang KC, Chang KJ, Shieh MJ, et al (2003) Comparison of medial-to-lateral versus traditional lateral-to-medial laparoscopic dissection sequences for resection of rectosigmoid cancers: randomized controlled clinical trial. World J Surg 27: 190–196. 12616435 10.1007/s00268-002-6437-y

[20] Veldkamp R, Gholghesaei M, Bonjer HJ, Meijer DW, Buunen M, et al (2004) Laparoscopic resection of colon Cancer: consensus of the European Association of Endoscopic Surgery (EAES). Surg Endosc 18: 1163–1185. 15457376 10.1007/s00464-003-8253-3

[21] Le Voyer TE, Sigurdson ER, Hanlon AL, Mayer RJ, Macdonald JS, et al (2003) Colon cancer survival is associated with increasing number of lymph nodes analyzed: a secondary survey of intergroup trial INT-0089. J Clin Oncol 21: 2912–2919. 12885809 10.1200/JCO.2003.05.062

[22] Chang GJ, Rodriguez-Bigas MA, Skibber JM, Moyer VA (2007) Lymph node evaluation and survival after curative resection of colon cancer: systematic review. J Natl Cancer Inst 99: 433–441. 17374833 10.1093/jnci/djk092

[23] Li JC, Leung KL, Ng SS, Liu SY, Lee JF, et al (2012) Laparoscopic-assisted versus open resection of right-sided colonic cancer—a prospective randomized controlled trial. Int J Colorectal Dis 27: 95–102. 10.1007/s00384-011-1294-5 21861071

[24] Han DP, Lu AG, Feng H, Wang PX, Cao QF, et al (2014) Long-term outcome of laparoscopic-assisted right-hemicolectomy with D3 lymphadenectomy versus open surgery for colon carcinoma. Surg Today 44: 868–874. 10.1007/s00595-013-0697-z 23989942

[25] Nakamura T, Onozato W, Mitomi H, Naito M, Sato T, et al (2009) Retrospective, matched case-control study comparing the oncologic outcomes between laparoscopic surgery and open surgery in patients with right-sided colon cancer. Surg Today 39: 1040–1045. 10.1007/s00595-009-4011-z 19997798

